# Bacterial Genetic Approach to the Study of Reactive Oxygen Species Production in *Galleria mellonella* During *Salmonella* Infection

**DOI:** 10.3389/fcimb.2021.640112

**Published:** 2021-03-01

**Authors:** Hanna D. Bismuth, Gaël Brasseur, Benjamin Ezraty, Laurent Aussel

**Affiliations:** Aix-Marseille Université, CNRS, Laboratoire de Chimie Bactérienne, Institut de Microbiologie de la Méditerranée, Marseille, France

**Keywords:** *Galleria mellonella*, *Salmonella enterica*, reactive oxygen species, host-pathogen interactions, biosensors

## Abstract

Over the last decade, an increasing number of reports presented *Galleria mellonella* larvae as an important model to study host-pathogen interactions. Coherently, increasing information became available about molecular mechanisms used by this host to cope with microbial infections but few of them dealt with oxidative stress. In this work, we addressed the role of reactive oxygen species (ROS) produced by the immune system of *G. mellonella* to resist against *Salmonella enterica*, an intracellular pathogen responsible for a wide range of infections. We confirmed that *Salmonella* was pathogen for *G. mellonella* and showed that it had to reach a minimal bacterial load within the hemolymph to kill the larvae. ROS production by *G. mellonella* was revealed by the virulence defects of *Salmonella* mutants lacking catalases/peroxiredoxins or cytoplasmic superoxide dismutases, both strains being highly sensitive to these oxidants. Finally, we used bacterial transcriptional fusions to demonstrate that hydrogen peroxide (H_2_O_2_) was produced in the hemolymph of *Galleria* during infection and sensed by *S. enterica*. In line with this observation, the H_2_O_2_-dependent regulator OxyR was found to be required for bacterial virulence in the larvae. These results led us to conclude that ROS production is an important mechanism used by *G. mellonella* to counteract bacterial infections and validate this host as a relevant model to study host-pathogen interactions.

## Introduction

The oxidative burst is one of the major mechanisms of the host innate immune system and the ability of pathogens to cope with this stress is often correlated with their virulence; that’s why studying such resistance mechanism is of primary importance to assess bacterial pathogenicity. *In vivo* assays are generally carried out in mammalian models, which are expensive, require significant expertise and a secure experimental environment. They also become ethically and socially controversial, leading to the emergence of alternative models. Among them, *Galleria mellonella* larva is of increasing interest. This lepidopteran was first used in the 1960s to test the virulence of a wide variety of microorganisms, from bacteria to fungi and viruses (Kurstak and Vega, 1968; Lysenko and Kucera, 1968; Younghusband and Lee, 1969). Over the last decade, it has become an attractive model in the field of host-pathogen interactions (Repizo et al., 2015; Cools et al., 2018; Pereira et al., 2018; Sciuto et al., 2018; Candela et al., 2019; Barros et al., 2019). And more recently, a high-throughput screening was carried out in *G. mellonella* to evaluate the synergy between antibiotics, human drugs and food additives on a various bacteria (Brochado et al., 2018).

Despite increasing information dealing with *Galleria mellonella* antibacterial mechanisms, the characterization of ROS production by the immune system remains elusive. In Lepidoptera, two pathways were proposed to produce free radicals: the humoral response through the production of melanin and the cellular response through phagocytosis and assembly of the NADPH oxidase (Sugumaran, 2002; Bergin et al., 2005). Oxidation reactions were first detected into free cells of *Galleria* hemolymph by Electron Spin Resonance spectroscopy but neither exogenous superoxide dismutase (SOD), nor phagocytosis activators were found to change the oxidation level (Slepneva et al., 1999). The Kavanagh group also demonstrated that the kinetics of phagocytosis and microbial killing were similar in *Galleria* hemocytes and human neutrophils (Bergin et al., 2005). Superoxide production and microbial killing were inhibited in the presence of an NADPH oxidase inhibitor, and immunoblotting of *G. mellonella* hemocytes with antibodies raised against human neutrophil *phox* proteins revealed the presence of proteins homologous to gp91^phox^ and p67^phox^ (Bergin et al., 2005). Nevertheless, these studies were conducted *ex vivo* and led to two open questions: (i) Does *Galleria* immune system produces ROS during bacterial infection and (ii) is this mechanism efficient to kill pathogens?

To answer these questions, we used *Salmonella enterica* serovar Typhimurium as a bacterial model. This facultative intracellular bacterium causes a wide range of infections and exhibits a broad host spectrum for various living organisms in which its virulence can be easily tested. Despite the fact that *Salmonella* is not a natural pathogen for *Galleria*, this bacteria was used for the first time in the host model *Galleria mellonella* in 1968 and showed to be pathogen for the larvae (Kurstak and Vega, 1968). After a gap of 45 years in literature, new sets of experiments using *Galleria* were conducted in the 2010s to characterize or confirm the role of *Salmonella* virulence factors, such as PhoQ activity and LPS (Bender et al., 2013), Rnases E and III (Viegas et al., 2013) or AcrB efflux function (Wang-Kan et al., 2017).

In the present work, we took advantage of the facile genetics approach and extensive literature of *Salmonella enterica* to address the implication of ROS produced by the immune system of *G. mellonella*. *S. enterica* produces an arsenal of detoxifying enzymes, which differ by their cellular location and substrate specificity. SODs allow the dismutation of superoxide $\left({\text{O}}_{2^{\cdot -}}\right)$ into hydrogen peroxide (H_2_O_2_). SodA and SodB are located in the bacterial cytoplasm whereas SodCI and SodCII are located the periplasm (Tsolis et al., 1995; Fang et al., 1999; Krishnakumar et al., 2004). Furthermore, three catalases and two peroxidases are involved in H_2_O_2_ degradation within the cytoplasm (Hébrard et al., 2009). Inactivation of these five genes yielded the HpxF mutant, which exhibits a severe survival defect within macrophages and mice (Hébrard et al., 2009). Therefore, *Salmonella* relies on its capacity to metabolize and to degrade ROS produced by the host to cope with oxidative stress.

In this study, we confirmed that *Salmonella* was a pathogen for *G. mellonella* and we showed that it had to reach a threshold inside the hemolymph to kill the host. Virulence defects of *Salmonella* mutants lacking either antioxidant defences or redox-activated regulators suggest that *Galleria* immune system has the capacity to produce ROS. This hypothesis was validated by *in vivo* biosensors assays which allowed us to conclude that H_2_O_2_ was produced in the hemolymph of *Galleria* during *Salmonella* infection.

## Method

### Bacterial Strains

The strains and plasmids used in this study are listed in **Table S1**. *Salmonella enterica* serovar Typhimurium ATCC 14028 and *Escherichia coli* MG1655 were used in this study. Deletions of genes were carried out using one-step λ Red recombinase chromosomal inactivation system (Datsenko and Wanner, 2000). In *Salmonella enterica*, deletions were transferred to the WT strain using P22 transduction procedures and verified by PCR. Heat inactivated bacteria were incubated 15 min at 65°C. Strains were grown at 37°C in lysogeny broth (LB) medium.

### Plasmids Construction

The cloning vector used to monitor gene expression was pFPV25, carrying promotorless *gfpmut3a* gene. The inserts carrying 300 bp upstream *ahpC* or *soxS* start codon were PCR-amplified from *S. enterica* 12023 by using the primers listed in **Table S2**. PCR products were digested using *XbaI* and *NdeI*, and cloned into pFPV25 vector, yielding P*ahpC-gfp* and P*soxS-gfp* plasmids (**Table S1**). All the inserts were verified by DNA sequencing. The cloning vector used to detect *Salmonella* cells within the hemolymph was pGBM2, carrying promotorless *mCherry* gene. An insert carrying 300 bp upstream *rpsM* was digested using *KpnI* and *HindIII*, and cloned into pGBM2 vector, yielding the P*rpsM-mCherry* plasmid (**Table S1**).

### Insects

*Galleria mellonella* larvae (Lepidoptera: Pyralidae, the Greater Wax Moth) (Sud-Est Appats, Queige, France) were stored in wood chips in the dark at 22°C. All larvae were 5 to 6 weeks old and weighed between 300 and 500 mg. They were used within 1 week.

### Bacterial Infection of *Galleria mellonella*

Bacterial strains were grown during 16 h in LB under microaerobic conditions (screwed tubes, 37°C without shaking). The cultures were washed and immediately diluted in phosphate-buffered saline (PBS, NaCl 137 mM, KCl 2.7 mM, Na_2_HPO_4_ 10 mM, KH_2_PO_4_ 1.8 mM, pH 7.4) to a final concentration of 10^6^ CFU/ml. *G. mellonella* larvae were incubated 16 h at 37°C before injection. 10 μl of the suspension containing 10^4^ CFU of bacteria was injected into the last proleg of the larvae. Injected larvae were incubated at 37°C and death was assessed 24, 48, and 72 h post-injection. Experiments were repeated three times using at least 20 larvae per group. Differences in survival between larvae injected with the WT strain and *Salmonella* mutants were determined by Kaplan-Meier analysis with log-rank test.

### Bacterial Viability in *Galleria mellonella*

Injections were carried out as described above and the hemolymph was collected as previously described (Candela et al., 2019). Briefly, 6, 12, 18, and 24 h post-injection, larvae were washed once in 70% ethanol and twice in PBS to minimize surface contaminants. The abdomen of the injected larvae was pricked with a sterile needle. 10 µl of hemolymph was collected with a pipette and incubated 5 min with Triton X-100 0.5% to release intracellular bacteria and to bring together the whole bacterial population (intracellular and free bacteria). Bacterial suspensions obtained after centrifugation were washed with PBS, serial-diluted in PBS, and spotted on LB agar plates. CFU were counted after 18 h at 37°C.

### Bacterial Viability *In Vitro*

Bacterial strains were grown overnight in LB, washed, and serial-diluted in PBS. After that, 5 µl of the different dilutions were spotted on LB agar plates with or without bovine liver catalase (2,000 U/plate; Sigma-Aldrich), hydrogen peroxide (20 or 50 µM), or paraquat (50 or 100 µM), a redox cycler which stimulates superoxide production. Catalase, hydrogen peroxide, and paraquat were purchased from Sigma-Aldrich (Lyon, France). CFU were counted after 18 h at 37°C.

### Flow Cytometry Analysis of Bacteria Extracted From Hemolymph

*G. mellonella* larvae were injected with 10^5^ bacteria carrying the constitutive P*rpsM-mCherry* plasmid (to identify *Salmonella* cells within the hemolymph) and either P*ahpC-gfp*, or P*soxS-gfp* inducible plasmids (to monitor oxidative stress). In addition, 20 µl of hemolymph were collected 30 and 60 min post-injection, diluted in an anticoagulant solution, lysed with Triton X-100 0.5% and fixed in paraformaldehyde 3.2% during 20 min (Stoepler et al., 2012). Bacteria were pelleted at 6.500 g for 5 min and resuspended in 500 µl Dulbecco’s phosphate-buffered saline (DPBS, NaCl 138 mM, KCl 2.7 mM, Na_2_HPO_4_ 8.1 mM, KH_2_PO_4_ 1.5 mM, pH 7.0–7.3). For flow cytometric analysis, bacteria were first gated for their size/granulosity (FSCxSSC), for singlets in order to remove multiple events, then for the mCherry fluorescence (FL3 615 ± 25 nm) and finally analysed for the expression level of GFP (FL1 525 ± 30 nm). A compensation was applied on the mCherry signal due to the GFP overlapping signal. Samples were run in the low-pressure mode (about 10,000 particles/s). Data were acquired with an S3e cells sorter (Biorad) using 488 and 561 nm lasers and data were analyzed and plotted using FlowJo v10.6.

## Results

### *Salmonella enterica* Proliferation and Virulence in *Galleria mellonella*

To assess *S. enterica*’s capacity to infect *G. mellonella*, injections of 10^4^ or 10^5^ bacteria per larva were carried out. Injecting 10^4^ bacteria/larva led to the survival of 40% of the population 24 h post-injection whereas injecting 10^5^ bacteria/larva killed almost all the larvae in the same period (**Figure S1A**). This result is in accordance with the DL_50_ previously found by others (Bender et al., 2013). No significant killing was observed with *Escherichia coli* K12 and *S. enterica* heat inactivated strains, leading to the conclusion that *Salmonella*, and not *E. coli*, exhibited an active virulence mechanism against *Galleria* (**Figure S1A**). To decipher the early stages of *Salmonella* invasion within *G. mellonella*, we measured bacterial colony-forming units (CFU) in the hemolymph 6, 12, 18 and 24 h post-injection of 10^4^ bacteria/larva. Killed larvae were observed from 12 h post-injection and the bacterial load upon death (BLUD) median value was found to be >10^8^ CFU/larva (**Figure S1B**). This parameter, often used in host/pathogen interactions, allows to determine the ability of the host to resist infection. Twenty-four hours post-injection, a large majority of alive larvae cleared all bacteria (**Figure S1B**). In line with the results presented above, 60% of the larvae were killed 24 h post-injection. These data indicate that *G. mellonella* larvae injected with 10^4^ *Salmonella* cells suffer a different fate and that a minimal bacterial load has to be reached in the hemolymph to kill the host.

### Antioxidant Enzymes Support *Salmonella* Virulence in *Galleria*

**Figure 1:**
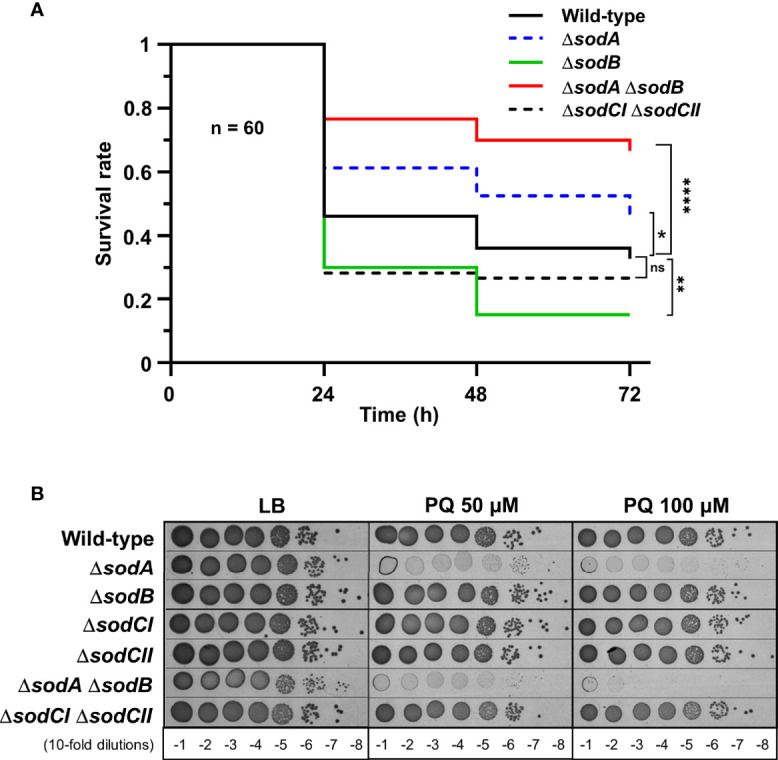
Cytoplasmic superoxide dismutases support *Salmonella* virulence in *Galleria*. **(A)** *Galleria mellonella* larvae were injected with 10 µl of solutions containing *Salmonella* WT, the singles mutants Δ*sodA* or Δ*sodB*, and the double mutants Δ*sodA* Δ*sodB* or Δ*sodCI* Δ*sodCII* at a concentration of 10^4^ bacteria/larva. Infections were repeated three times using 20 larvae per group. The survival curves were compared by log-rank. ns, not significant; *P ≤ 0.05; **P ≤ 0.01; ****P ≤ 0.0001 (Mantel-Cox test). **(B)** The WT, Δ*sodA*, Δ*sodB*, Δ*sodCI*, Δ*sodCII*, Δ*sodA* Δ*sodB*, and Δ*sodCI* Δ*sodCII* strains were grown in LB, serial diluted as indicated and spotted on LB agar plates or increasing concentrations of paraquat (PQ). The results are representative of three independent experiments.

**Figure 2:**
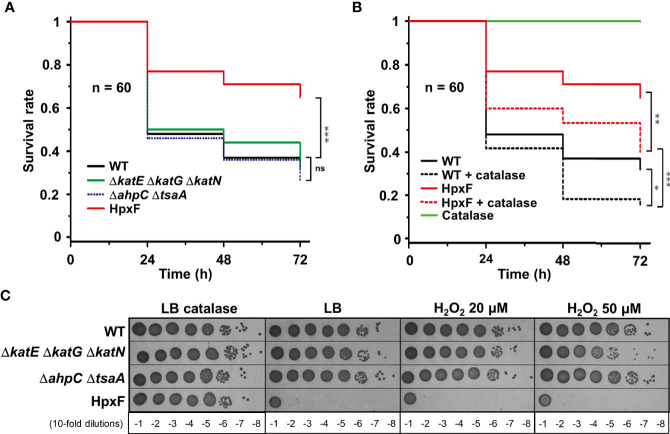
Sensitivity of *Salmonella* catalases/peroxiredoxins mutants reveal ROS production by *Galleria*. **(A, B)** *Galleria mellonella* larvae were injected with 10 µl of solutions containing *Salmonella* wild-type, Δ*katE* Δ*katG* Δ*katN*, Δ*ahpC* Δ*tsaA*, and HpxF strains at a concentration of 10^4^ bacteria/larva. When indicated, catalase is used at a concentration of 4 mg/kg of larva. Infections were repeated three times using 20 larvae per group. The survival curves were compared by log-rank. ns, not significant; *P ≤ 0.05; **P ≤ 0.01; ***P ≤ 0.001 (Mantel-Cox test). **(C)** The wild-type, Δ*katE* Δ*katG* Δ*katN*, Δ*ahpC* Δ*tsaA*, and HpxF strains were grown in LB, serial diluted as indicated and spotted on LB agar plates with or without catalase or increasing concentrations of hydrogen peroxide (H_2_O_2_).

To investigate *G. mellonella* superoxide production in antibacterial defence, we have constructed *Salmonella* mutants inactivated in cytoplasmic (*sodA* and *sodB*) or periplasmic (*sodCI* and *sodCII*) SOD-encoding genes. *Galleria* larvae injected with the Δ*sodA* Δ*sodB* mutant exhibited a survival rate two-fold higher than those injected with the WT strain, whereas no significant difference was observed between the Δ*sodCI* Δ*sodCII* mutant and the WT (**Figure 1A**). These results were in accordance with the sensitivity of these mutants to paraquat, a superoxide generator, i.e., the Δ*sodA* Δ*sodB* mutant was highly sensitive to paraquat whereas the Δ*sodCI* Δ*sodCII* mutant was not (**Figure 1B**). Surprisingly, we observed that the Δ*sodB* mutant was slightly more virulent than the WT strain (**Figure 1A**). Next, we observed that a *Salmonella* strain lacking all H_2_O_2_ degrading activities (catalases KatE, KatG, and KatN, and peroxiredoxins AhpC and TsaA), referred to as HpxF, exhibited an attenuated virulence in *G. mellonella* (**Figure 2A**). Larvae injected with catalase (Δ*katE* Δ*katG* Δ*katN*) or peroxiredoxin (Δ*ahpC* Δ*tsaA*) mutants showed a survival rate comparable to those injected with the WT strain (**Figure 2A**). Interestingly, co-injection of exogenous catalase with the HpxF mutant and the WT strain strongly decreased *Galleria*’s survival rate, indicating that oxidative stress fully participates to *Galleria* immune response (**Figure 2B**). As expected, the HpxF mutant was particularly sensitive to H_2_O_2_ whereas others were not (**Figure 2C**). Nevertheless, no growth defect was observed in liquid LB medium under microaerobic conditions for the HpxF and for the Δ*sodA* Δ*sodB* mutants compared to the wild-type strain (**Figure S2**). Together, these observations highlight the importance of ROS produced by *Galleria* immune system to counteract *Salmonella* infection.

### *Salmonella* Sensed H_2_O_2_ Within *Galleria* Hemolymph

**Figure 3:**
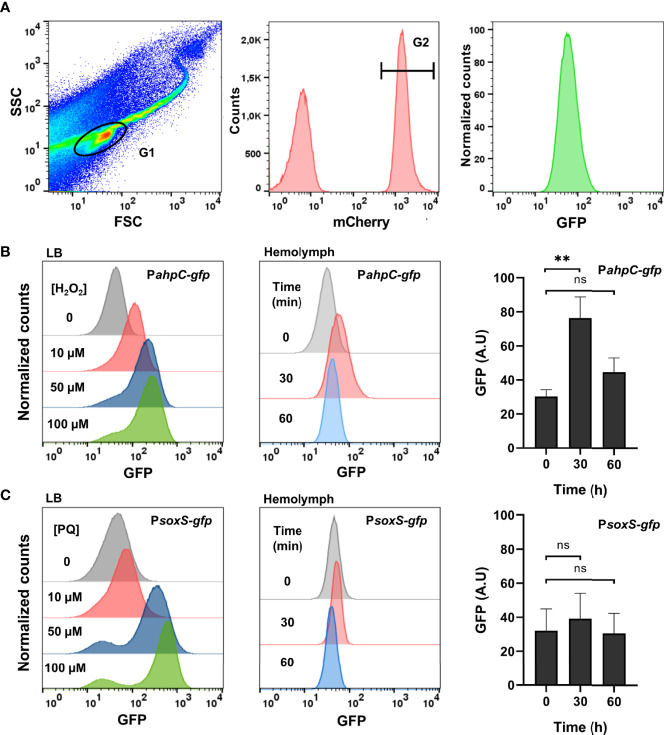
*Salmonella* experiences hydrogen peroxide within *Galleria* hemolymph. **(A)** Flow cytometry analysis. The first gate (G1) was based on size and granularity (FSC x SSC), outlining the bacterial population perimeter. The second gate (not shown) was intended to remove doublet events. *Salmonella* cells carried a constitutive mCherry plasmid and this population was gated in G2 by their red fluorescence. Finally, the GFP fluorescence of the bacterial population (G2) was measured. **(B, C)** A WT strain carrying the P*ahpC-gfp* **(B)** and the P*soxS-gfp* **(C)** fusions were grown in LB. Increasing concentrations of H_2_O_2_ **(B)**, left and paraquat **(C)**, left were added and the florescence was measured by flow-cytometry 30 min post-treatment. *Galleria* larvae were injected with a WT strain carrying the P*ahpC-gfp* **(B)**, center and right and the P*soxS-gfp* **(C)**, center and right fusions. Bacteria were extracted and analyzed from the hemolymph 30 and 60 min post-injection. The fluorescence was measured by flow cytometry and the raw data are shown on these panels where a representative experiment (center) and the quantification of three independent experiments (right) are presented. Asterisks indicate a statistically significant difference between two infection times. ns, not significant; **P ≤ 0.01 (Dunnett test).

To characterize the ROS produced by *Galleria* and sensed by *Salmonella*, injection of larvae was carried out with bacterial strains carrying either the H_2_O_2_-inducible P*ahpC-gfp* fusion or the $O_{2^{\cdot -}}$-inducible P*soxS-gfp* fusion. Bacteria were collected from the hemolymph and analysed by flow cytometry. They were first sorted for *mCherry* expression as all injected *Salmonella* cells carried the P*rpsM-mCherry* constitutive fusion (**Figure 3A**). GFP intensity was then measured as all injected *Salmonella* cells carried the P*ahpC-gfp* or P*soxS-gfp* fusions, reflecting the level of ROS experienced by *Salmonella* within the host. *In vitro* controls were first carried out and showed that addition of 10 to 100 µM H_2_O_2_ in the LB medium led to a dose-dependent induction of the P*ahpC-gfp* fusion 30 min post-treatment (**Figure 3B**, left). A similar induction was observed with the P*soxS-gfp* fusion treated with increasing concentrations of paraquat in LB (**Figure 3C**, left). These bacterial strains were next injected in *G. mellonella* and 30 min post-injection of larvae, a 2.5-fold induction of the P*ahpC-gfp* fusion was measured whereas the level of the P*soxS-gfp* fusion did not change significantly (**Figures 3B, C**, right). The fluorescence of the P*ahpC-gfp* fusion recovered its basic level 60 min post-injection, indicating a possible decrease of H_2_O_2_ production by *Galleria* immune system and/or ROS degradation by *Salmonella* within this interval of time. Altogether, these results show that an H_2_O_2_ burst was generated by *Galleria* immune system and sensed by *Salmonella* just after infection.

### The H_2_O_2_-Dependent Activator OxyR Is Required for *Salmonella* Full Virulence in *G. mellonella*

**Figure 4:**
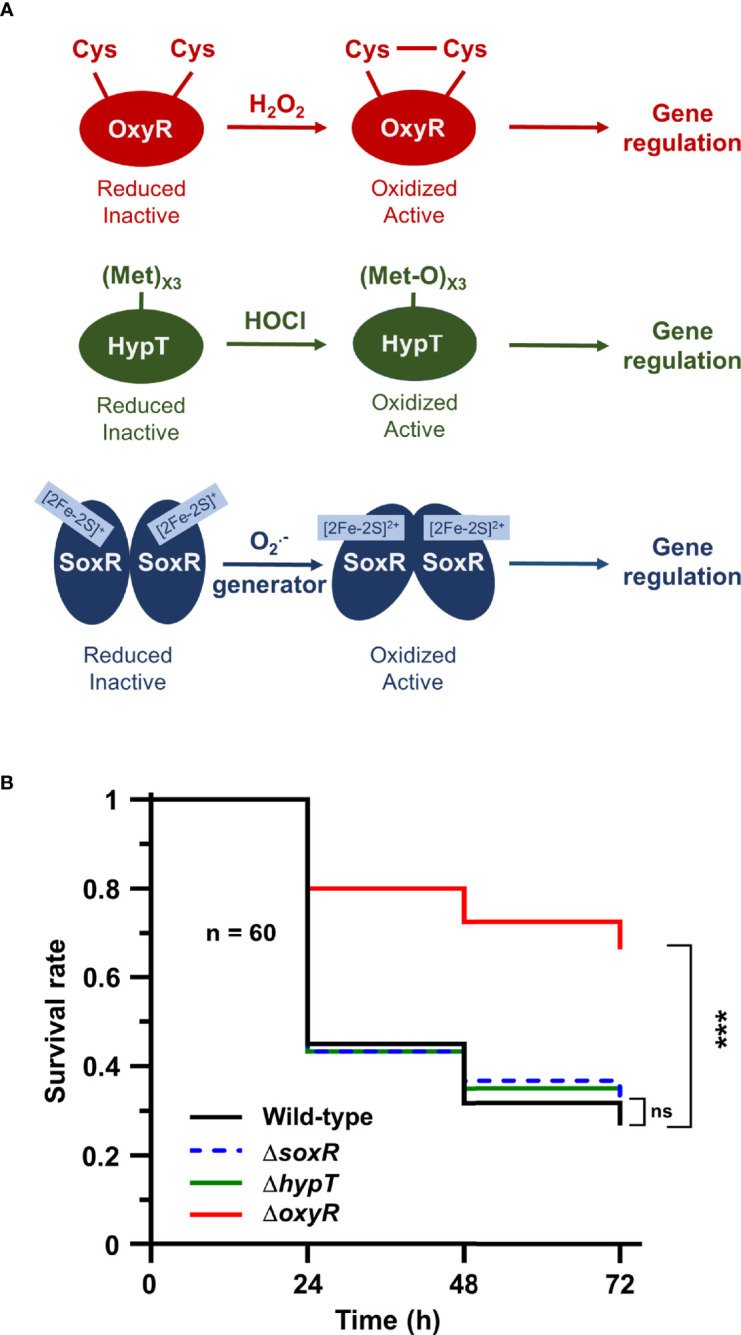
The OxyR regulator is required for *Salmonella* virulence in *Galleria*. **(A)** Models of OxyR, HypT, and SoxR activation by hydrogen peroxide (H_2_O_2_), hypochlorous acid (HOCl), and redox-recycling drugs or superoxide generator $\left(O_{2^{\cdot -}}\right)$, respectively. **(B)** *Galleria mellonella* larvae were injected with 10 µl of suspensions containing *Salmonella* WT and the Δ*oxyR*, Δ*hypT* and Δ*soxR* mutants at a concentration of 10^4^ bacteria/larva. Infections were repeated three times using 20 larvae per group. The survival curves were compared by log-rank. ns, not significant; ***P ≤ 0.001 (Mantel-Cox test).

Next, we addressed the role of oxidative stress-dependent transcriptional regulators in *Salmonella* to trigger adaptive responses inside the host. We focused on three of them: OxyR dependent upon H_2_O_2_ concentration, HypT activated by hypochlorite acid (HOCl), and SoxR which responds to redox cycling drugs and $O_{2^{\cdot -}}$ (**Figure 4A**). *Galleria* larvae injected with the Δ*oxyR* mutant exhibited a survival rate two-fold higher than the WT strain (**Figure 4B**). Conversely, no virulence defects were observed for the Δ*hypT* and the Δ*soxR* mutants (**Figure 4B**). These results did not suggest any major role for HypT and SoxR in *Salmonella* adaptive response during *Galleria* infection and highlight the importance of the H_2_O_2_-activated regulator OxyR in this process.

## Discussion

In this study, we showed that *Salmonella* strains inactivated for catalases/peroxiredoxins and cytoplasmic superoxide dismutases exhibited reduced virulence in *G. mellonella*, revealing oxidative stress production by the larvae. Moreover, induction of the P*ahpC-gfp* fusion in the hemolymph during the first stage of the infection indicated that an H_2_O_2_ burst was generated by *Galleria*. These results demonstrate that oxidative stress is an important immune mechanism used by *Galleria* to resist microbial infections.

The periplasmic SodCI and SodCII enzymes were previously found to be important for virulence in mice whereas cytoplasmic SodA was shown to be dispensable (Tsolis et al., 1995; Fang et al., 1999; Krishnakumar et al., 2004). In the present study, we found that the cytoplasmic SODs are required for *Salmonella* full virulence in *Galleria* and that the periplasmic SODs are dispensable. Therefore, *Galleria* can be used as a particularly relevant model to highlight bacterial defence systems whose importance could have been underestimated so far.

Superoxide anion production by *Galleria* was previously documented by *ex vivo* experiments and one could postulate that it should be sensed by *Salmonella* (Bergin et al., 2005). Nevertheless, our *in vivo* experiments did not reveal any activation of the $O_{2^{\cdot -}}$ biosensor located in *Salmonella* cytoplasm. Charged molecules cannot cross bacterial membranes and $O_{2^{\cdot -}}$ is rapidly dismutated into H_2_O_2_, either enzymatically or spontaneously, explaining at least partially the modest activation of the P*soxS-gfp* fusion. In addition, the half-life of superoxide anion was estimated to be about 5 s at physiological pH (Marklund, 1976). But we can’t exclude the presence of $O_{2^{\cdot -}}$ in the bacterial cytoplasm as the reduced virulence of the Δ*sodA* Δ*sodB* mutant strongly supports the importance of superoxide. We can hypothesize that *Galleria* produced oxidative stress in response to *Salmonella* invasion, $O_{2^{-}}$ being rapidly dismutated into H_2_O_2_ and targeted to the bacterial cytoplasm as demonstrated by the induction of the P*ahpC-gfp* fusion 30 min post-injection. A pervious study has reported the production of H_2_O_2_ in the hemolymph of *Galleria* in the first hours after injection of *Bacillus thuringiensis* (Komarov et al., 2006). Interestingly, a similar observation was previously made in mouse macrophages where an H_2_O_2_ burst was detected 45 min after *Salmonella* infection (Aussel et al., 2011). We did not investigate the role of phagocytosis during the infection but we showed that an H_2_O_2_ burst was produced by *Galleria* and sensed by *Salmonella* 30* min* post-infection. This correlation between *Galleria mellonella* and mice is an additional argument to validate this insect as a relevant alternative model to study host-pathogen interactions. Additional studies will be required to investigate the distribution of intracellular and free bacteria within the hemolymph.

Finally, we have assayed the importance of adaptive responses in *Salmonella* through the involvement of different oxidative stress-activated regulators. During the infection of *Galleria*, the HOCl-sensing regulator HypT appeared to be dispensable. This result is in accordance with the absence in *Galleria* of the major HOCl-producing enzyme, the myeloperoxidase (MPO). Indeed, cytochemistry and immunodetection analysis failed to detect MPO in the hemocytes of the larvae (Chain and Anderson, 1983; Fallon et al., 2011). All these observations suggest that *Galleria*’s hemocytes don’t produce HOCl. Moreover, insects were shown to synthesize a dual oxidase (DUOX) able to catalyse HOCl production in the epithelial cells of its gut (Kim and Lee, 2014). But *Salmonella* has never been in contact with epithelial cells in any of our experiments. Future work using dedicated HOCl reporters (Stocker et al., 2017) might solve this issue.

OxyR was identified as an important regulator to allow the success of *Salmonella* infection. This transcriptional activator is dependent upon H_2_O_2_ and can activate a regulon composed of more than 30 genes, most of them encoding antioxidant enzymes such as catalases, peroxiredoxins or thioredoxins (Zheng et al., 2001). We showed that an *oxyR* mutant was poorly virulent during *Galleria* infection, conferring to the regulator encoded by this gene a key role in detecting low H_2_O_2_ levels and triggering bacterial adaptive mechanisms to cope with oxidative stress. Moreover, a mutant unable to degrade H_2_O_2_ was found to be attenuated during the larvae infection. We also showed that *Salmonella* experienced H_2_O_2_ produced by *Galleria*. Taken together, our results highlight the importance of H_2_O_2_ in the hemolymph of *Galleria* to eradicate pathogens.

Like mammalian models, insects developed a wide variety of mechanisms to resist against pathogens, among which oxidative stress. Our findings showed that *Galleria mellonella* produces ROS to defend against *Salmonella enterica* infection and might be useful to complete the characterization of the immune system of this host, which appears to be a suitable model to study bacterial pathogenicity.

## Data Availability Statement

The raw data supporting the conclusions of this article will be made available by the authors, without undue reservation.

## Author Contributions

Conceptualization: BE and LA. Methodology: HB, GB, BE, and LA. Investigation: HB, GB, BE, and LA. Writing-original draft preparation: HB, GB, BE, and LA. All authors contributed to the article and approved the submitted version.

## Funding

This work was supported by grants from the Agence Nationale de la Recherche (ANR) (#ANR-16-CE11-0012-02 METOXIC), the Centre National de la Recherche Scientifique (CNRS) (#PICS-PROTOX), and Aix-Marseille Université.

## Conflict of Interest

The authors declare that the research was conducted in the absence of any commercial or financial relationships that could be construed as a potential conflict of interest.
