# Supplementary material for: Bacterial Genetic Approach to the Study of Reactive Oxygen Species Production in Galleria mellonella During Salmonella Infection
Source: Front Cell Infect Microbiol. 2021 Mar 1;11:640112. doi: 10.3389/fcimb.2021.640112 (PMC7957066; doi:10.3389/fcimb.2021.640112)
Supplement: Supplementary file 1 [file DataSheet_1.pdf]

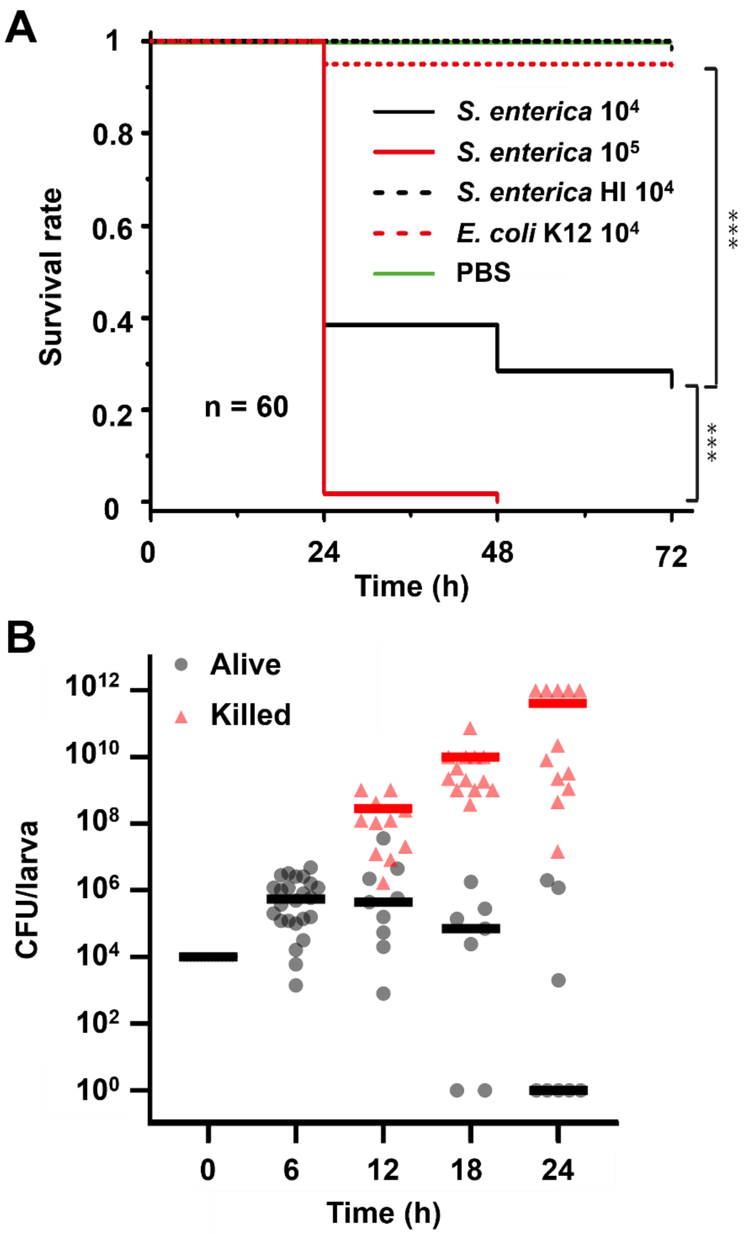

Figure S1: *Salmonella enterica* virulence in *Galleria mellonella*.

A. *Galleria mellonella* larvae were injected with 10 µl of a solution containing 10^4 or 10^5 *Salmonella* cells, heat inactivated *Salmonella* (HI) (10^4), *Escherichia coli* K12 (10^4) or PBS. Kaplan-Meier plots show larvae survival over 72 hours. Experiments were repeated three times using 20 larvae per group. The survival curves were compared by log-rank. ***P ≤ 0.001 (Mantel-Cox test). **B.** *Galleria* larvae were injected with 10^4 *Salmonella* cells. 6, 12, 18 and 24 hours post-injection, the hemolymph was removed from 24 larvae for bacterial counts on LB agar plates. The grey dots indicate CFU counted from alive larvae and the black bars represent the median value. The red triangles indicate CFU from killed larvae and the red bars represent the median value.

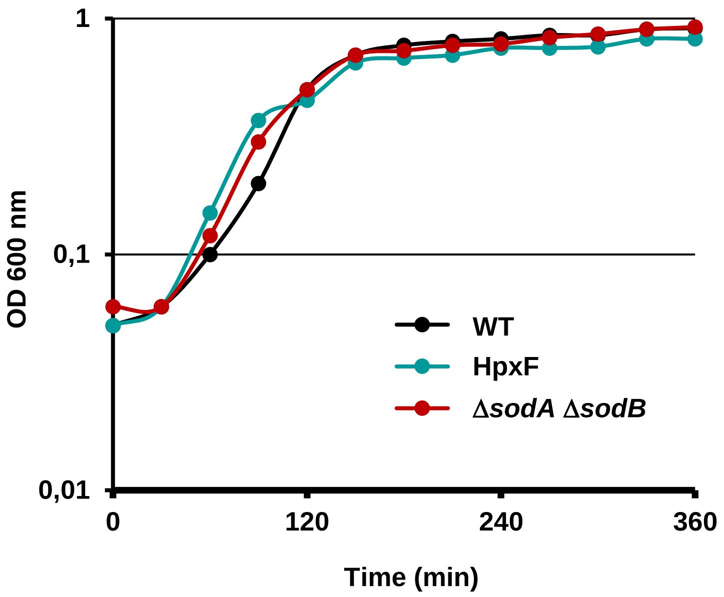

Figure S2: Growth of three *Salmonella enterica* strains under microaerobic conditions. *Salmonella enterica* wild-type (black circles), the HpxF (green circles) and the Δ*sodA* Δ*sodB* (red circles) cells were grown in LB medium in microaerobic conditions during 16 hours. The cells were then diluted in LB medium to an OD_{600} of 0.05, and microaerobic growth was monitored at 600 nm without shaking at 37°C.

Table S1. List of strains and plasmids used in this study.

Strain	Genotype and description	Source
Salmonella enterica 14028	*Salmonella enterica* serovar Typhimurium 14028 (wild-type)	Laboratory collection
Δ*sodA*	14028 Δ*sodA*::Cm^r	This study
Δ*sodB*	14028 Δ*sodB*::Cm^r	This study
Δ*sodA* Δ*sodB*	14028 Δ*sodA* Δ*sodB*::Cm^r	This study
Δ*sodCI*	14028 Δ*sodCI*::Cm^r	(Uzzau et al., 2002)
Δ*sodCII*	14028 Δ*sodCII*::Kan^r	(Uzzau et al., 2002)
Δ*sodCI* Δ*sodCII*	14028 Δ*sodCI*::Cm^r Δ*sodCII*::Kan^r	This study
Δ*katE* Δ*katG* Δ*katN*	14028 Δ*katE* Δ*katG* Δ*katN*::Kan^r	(Hébrard et al., 2009)
Δ*ahpC* Δ*tsaA*	14028 Δ*ahpCF* Δ*tsaA*::Kan^r	(Hébrard et al., 2009)
HpxF	14028 Δ*katE* Δ*katG* Δ*katN* Δ*ahpCF*::Kan^r Δ*tsaA*::Cm^r	(Hébrard et al., 2009)
Δ*oxyR*	14028 Δ*oxyR*::Tn10 (Tet^r)	(Elgrably-Weiss et al., 2002)
Δ*hypT*	14028 Δ*hypT*::Cm^r	This study
Δ*soxR*	14028 Δ*soxR*::Cm^r	This study
Escherichia coli K-12	*Escherichia coli* K-12 MG1655	Laboratory collection

**Plasmids**		
P*ahpC-gfp*	pFPV25 derivative carrying the *ahpC* promoter - Ap^r	(Aussel et al., 2011)
P*soxS-gfp*	pFPV25 derivative carrying the *soxS* promoter - Ap^r	This study
P*rpsm-mCherry*	pGBM2 derivative carrying the *rpsM* promoter - Kan^r	This study

References of this section

Aussel, L., Zhao, W., Hébrard, M., Guilhon, A.-A., Viala, J. P. M., Henri, S., et al. (2011). Salmonella detoxifying enzymes are sufficient to cope with the host oxidative burst. *Mol. Microbiol.* 80, 628–640. doi:10.1111/j.1365-2958.2011.07611.x.

Elgrably-Weiss, M., Park, S., Schlosser-Silverman, E., Rosenshine, I., Imlay, J., and Altuvia, S. (2002). A Salmonella enterica Serovar Typhimurium hemA Mutant Is Highly Susceptible to Oxidative DNA Damage. *J. Bacteriol.* 184, 3774–3784. doi:10.1128/JB.184.14.3774-3784.2002.

Hébrard, M., Viala, J. P. M., Méresse, S., Barras, F., and Aussel, L. (2009). Redundant hydrogen peroxide scavengers contribute to Salmonella virulence and oxidative stress resistance. *J. Bacteriol.* 191, 4605–4614. doi:10.1128/JB.00144-09.

Uzzau, S., Bossi, L., and Figueroa-Bossi, N. (2002). Differential accumulation of Salmonella [Cu, Zn] superoxide dismutases SodCI and SodCII in intracellular bacteria: correlation with their relative contribution to pathogenicity. *Mol. Microbiol.* 46, 147–156. doi:10.1046/j.1365-2958.2002.03145.x.

Table S2. List of oligonucleotides.

Name	Sequence
P*ahpC* fw	CCCTCTAGAGTAATGTAGAGCGCAACACTT
P*ahpC* rev	CCCCATATGTACTTCCTCCGTGTTTTCGTT
P*soxS* fw	CCCTCTAGATTGCCAGCGGGATGCCGATAC
P*soxS* rev	CCCCATATGGTTGCCTCTTAAACAGTATTC
sodA fw	GAACCAACTGCTTACGCGGCG
sodA rev	CATCTGCTGCTCCTTACGCGG
sodB fw	TTGCGCCACCGCTCAATTTGC
sodB rev	GGCGTCTCCTCTATTCTATCCC
soxR fw	CATCAATCCATTCGATAAGGG
soxR rev	GACGTCGGGGGAAACCCTCCTG
hypT fw	CCCGCTAGTTTCAGGCTGCCCC
hypT rev	CCGATGATTGATTCCACAGCGTCGC
